# Environmental Exposure and Long-Term Mortality After Coronary Artery Bypass Grafting: A Multicenter Cohort Study Beyond Traditional Risk Factors

**DOI:** 10.3390/toxics14060482

**Published:** 2026-05-31

**Authors:** Tomasz Urbanowicz, Sleiman Sebastian Aboul-Hassan, Krzysztof Skotak, Maria Luszczyn, Łukasz Moskal, Jakub Bratkowski, Mariusz Kowalewski, Jarosław Bartkowski, Bartłomiej Perek, Mirosław Wilczyński, Krzysztof J. Filipiak, Krzysztof Bartuś, Romuald Cichoń, Marek Jemielity

**Affiliations:** 1 Department of Cardiac Surgery and Transplantology, Poznan University of Medical Sciences, 61-848 Poznan, Poland; 2 Department of Cardiac Surgery, MEDINET Heart Center Ltd., 67-100 Nowa Sol, Poland; 3 Department of Cardiac Surgery and Interventional Cardiology, Medical University of Zielona Gora, 65-046 Zielona Gora, Poland; 4 Institute of Environmental Protection, National Research Institute, 01-045 Warsaw, Poland; 5 Department of Cardiac Surgery, Center of Postgraduate Medical Education, Central Clinical Hospital of the Ministry of Interior, 02-507 Warszawa, Poland; 6 Department of Cardiac Surgery, Central Clinical Hospital, Medical University of Lodz, 92-213 Lodz, Poland; 7 The Centre of Postgraduate Medical Education, 99/103 Marymoncka Street, 01-813 Warsaw, Poland; 8 Department of Cardiac Surgery and Transplantation, Jagiellonian University Medical College, 31-202 Krakow, Poland

**Keywords:** CABG, PM_10_, NO_2_

## Abstract

**Background:** Ambient air pollution is an established cardiovascular risk factor; however, its impact on long-term outcomes after coronary artery bypass grafting (CABG) remains insufficiently defined. We aimed to evaluate whether chronic exposure to air pollutants may influence long-term mortality following surgical revascularization. **Methods:** In this multicenter retrospective cohort study, 1033 consecutive patients undergoing CABG with BIMA (bilateral internal mammary arteries) grafting were analyzed with a median follow-up of 8.1 years. Individual exposure to nitrogen dioxide (NO_2_), particulate matter ≤10 μm (PM_10_), and ≤2.5 μm (PM_2.5_) was estimated based on residential data. Multivariable Cox proportional hazards models were used to assess associations with long-term mortality. Model performance was evaluated using receiver operating characteristic (ROC) analysis, while incremental prognostic value was quantified using net reclassification improvement (NRI) and integrated discrimination improvement (IDI). Kaplan–Meier analyses were performed using data-driven thresholds and model-based risk stratification. **Results:** During follow-up, 220 deaths (21.1%) occurred. In multivariable analysis, both NO_2_ and PM_10_ were associated with increased mortality (NO_2_: HR 2.70 per 10 μg/m^3^, 95% CI 2.03–3.59; PM_10_: HR 2.73 per 10 μg/m^3^, 95% CI 1.94–3.83; both *p* < 0.001), whereas PM_2.5_ was not significant. The clinical model demonstrated moderate discrimination (AUC 0.73), which improved significantly after inclusion of pollution variables (AUC 0.84; ΔAUC 0.11). Reclassification analysis showed substantial improvement (NRI 0.42, *p* < 0.001; IDI 0.11, *p* < 0.001). Kaplan–Meier analysis confirmed enhanced risk stratification, with a hazard ratio of 2.70 for the clinical model and 7.02 for the combined clinical and pollution model (both *p* < 0.001). **Conclusions:** In this retrospective cohort of patients undergoing CABG with BIMA grafting, higher long-term residential exposure to NO_2_ and PM_10_ was associated with greater all-cause mortality after adjustment for measured clinical and procedural factors. These findings support further investigation of environmental exposure as a prognostic marker in surgically treated coronary disease, pending external validation and more granular control for contextual confounding. These findings suggest that environmental exposure may represent a relevant component of long-term risk stratification, although confirmation in large-volume cohorts is required.

## 1. Introduction

Air pollution has emerged as a major, non-traditional associated factor of cardiovascular risk, contributing substantially to global morbidity and mortality [1,2,3,4,5,6,7]. Exposure to particulate matter and nitrogen oxides has been consistently linked to endothelial dysfunction, vascular inflammation, and accelerated atherosclerosis, thereby increasing the risk of both incident and recurrent cardiovascular events [8].

Patients treated with coronary artery bypass grafting (CABG) represent a distinct clinical phenotype in which obstructive coronary disease has been surgically corrected, yet long-term risk remains substantial [9]. In this setting, prognosis is traditionally interpreted through the lens of patients’ characteristics, ventricular function, and procedural data [10,11]. The potential novel markers and hematological parameters obtained from peripheral blood were analyzed as predictors of long-term outcomes after CABG in previous analyses [12,13,14]. However, this framework implicitly assumes that cardiovascular risk is predominantly intrinsic to the patient, with limited consideration of the external environment as a persistent modifier of disease trajectory.

This assumption may be overly simplistic. Environmental exposures, unlike conventional risk factors, act continuously and systemically, potentially influencing vascular biology long after revascularization has been achieved. Experimental and epidemiological studies have demonstrated that air pollution promotes chronic low-grade inflammation, oxidative stress, autonomic imbalance, and prothrombotic states—processes that are not directly addressed by surgical intervention [15,16]. As a result, even in anatomically revascularized patients, environmental exposures may sustain or reinitiate pathophysiological pathways leading to adverse outcomes [17,18].

A previous report [19] associated increased cardiovascular mortality with ambient air pollution, with the annual 5090 (95% CI: 4253–5888) attributable deaths mainly focusing on particulate matter with an aerodynamic diameter ≤2.5 μm (PM_2.5_). In the Swiss National Cohort analysis [20], natural mortality was associated with a 1.04-fold (1.03–1.04) increase per interquartile range (IQR) in NO_2_ for residential exposure. Long-term exposure to nitrogen dioxide (NO_2_) [21], a marker of traffic-related pollution, has been linked to elevated cardiovascular death risk, with meta-analyses reporting hazard ratios (HRs) of approximately 1.05–1.11, reflecting the contribution of oxidative stress, systemic inflammation, and endothelial dysfunction. Similarly, PM_10_ exposure is associated with both short- and long-term cardiovascular mortality. Short-term increases in PM_10_ are linked to acute rises in cardiovascular deaths (RR ≈ 1.006–1.01 per 10 μg/m^3^), while long-term exposure shows stronger associations, with HRs typically around 1.10–1.20 for higher exposure levels [22]. These pollutants promote atherosclerosis progression, trigger arrhythmias, and increase thrombogenicity, thereby contributing to fatal cardiovascular events. Given their widespread presence, NO_2_ and PM_10_ represent major modifiable environmental determinants of cardiovascular mortality at the population level.

To our knowledge, this is the first study to specifically evaluate the long-term prognostic impact of ambient air pollution in patients undergoing CABG with BIMA grafting. Furthermore, this represents the first systematic assessment of this association in a Central European (Polish) population. Notably, our findings diverge from prior literature by showing no association for PM_2.5_, warranting further mechanistic and epidemiological consideration.

In this context, we conducted a retrospective analysis to evaluate the association between long-term exposure to nitrogen dioxide (NO_2_), particulate matter ≤ 10 μm (PM_10_), and particulate matter ≤ 2.5 μm (PM_2.5_), and long-term mortality after CABG [23]. We hypothesized that ambient air pollution may be regarded as a possible predictor of long-term prognosis in this population, beyond established clinical and procedural risk factors.

## 2. Methods

### 2.1. Study Sample

Of 1042 consecutive patients screened between January 2006 and December 2025, 9 were excluded due to: missing residential exposure data (n = 5), incomplete covariate data (n = 3), and loss to follow-up (n = 1).

Because the enrollment period spanned nearly two decades, secular changes in surgical practice, perioperative care, secondary prevention strategies, and environmental exposure patterns may have influenced outcomes. Although participating centers maintained consistent surgical protocols for BIMA grafting, residual temporal confounding cannot be excluded.

**Figure 1:**
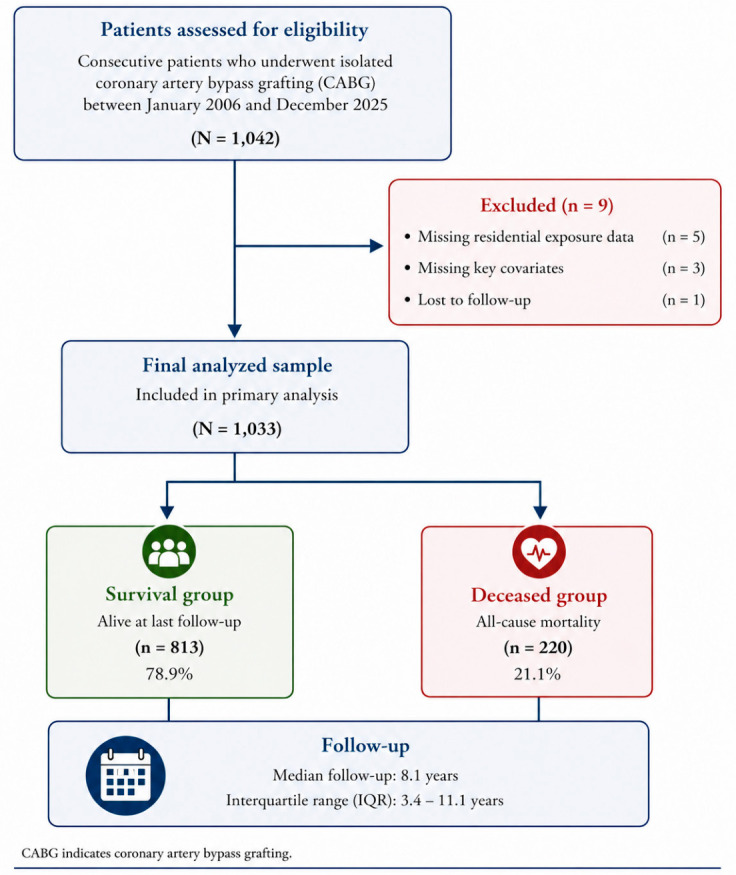
Flow chart: Patients’ enrolment.

The study enrolled patients who underwent surgical arterial revascularization, defined as the use of two internal mammary arteries for coronary artery bypass grafting, resulting in a final analytic sample of 1033 patients; a study flow diagram is provided in Figure 1. The two participating centers represent distinct geographic regions (western/south and western/central Poland), enhancing environmental variability and external validity.

### 2.2. Exposure Assessment

Air pollution exposure was defined using annual mean concentrations in the residential areas of: NO_2_ (nitrogen dioxide), PM_2.5_ (particulate matter up to 2.5 microns in diameter), and PM_10_ (particulate matter up to 10 microns in diameter). Exposure was defined as the time-weighted mean concentration over the follow-up period, accounting for survival time or censoring.

Air pollution data were obtained from the National Environmental Monitoring System (Chief Inspectorate of Environmental Protection, Poland), based on ground monitoring stations complemented by validated spatial interpolation models (kriging-based). Exposure estimates were assigned at the residential municipality level (spatial resolution: ~1–5 km grid). Individual exposure was calculated as the time-weighted mean concentration over the entire follow-up period, accounting for survival time or censoring. Residential addresses were geocoded and linked to the nearest monitoring grid using GIS-based mapping. The presented method is one of the assessment techniques for environmental exposure analysis [24,25,26].

Residential mobility during follow-up could not be systematically assessed; therefore, exposure estimates were based on the recorded baseline residential address. As a result, temporal changes in individual exposure related to relocation were not captured. Additionally, exposure estimates reflected outdoor residential exposure only and did not account for occupational exposure, commuting patterns, or indoor air pollution.

The use of municipality-level exposure assignment introduces the possibility of non-differential exposure misclassification, which may have attenuated or distorted observed associations.

### 2.3. Data Quality, Missing Data, Multicenter Consistency

Data entry was independently verified by two investigators. Outliers were assessed using interquartile range criteria and cross-checked with source records.

The proportion of missing data was <2% for all variables; complete-case analysis was applied.

Data harmonization between centers was ensured using standardized definitions and centralized data cleaning.

Outcome: The primary endpoint was long-term all-cause mortality.

### 2.4. Covariates

Models were adjusted for demographics (age, sex, body mass index (BMI)), clinical comorbidities (diabetes, hypertension, renal impairment, peripheral artery disease (PVD), prior cardiovascular adverse events (CVAEs)), cardiac status (left ventricular ejection fraction (LVEF), and procedural variables (off-pump coronary artery bypass grafting (OPCAB), number of grafts). Kidney impairment was defined as a glomerular filtration rate below 60 mL/min/1.73 m^2^.

Hypertension and diabetes were defined based on documented diagnosis and/or active pharmacological treatment.

CVAEs were defined according to established clinical criteria (ESC guidelines), including documented myocardial infarction or cerebrovascular events [27].

Postoperative complications were excluded from the primary model.

Importantly, several potentially relevant contextual and behavioral variables were unavailable in the present dataset, including smoking status, socioeconomic status, educational attainment, occupational exposure, physical activity, dietary patterns, medication adherence, and urban–rural residence. These factors are known to correlate both with environmental exposure and cardiovascular outcomes and therefore represent potential sources of residual confounding. Consequently, the present analyses should be interpreted as evaluating associations rather than causal effects.

### 2.5. Statistical Analysis

Continuous variables were presented as medians with interquartile ranges and compared using appropriate non-parametric tests. Categorical variables were expressed as counts and percentages.

Time-to-event analysis was performed using Cox proportional hazards regression. Univariable models were initially constructed for all candidate variables, followed by multivariable models including demographic, clinical, and procedural covariates. Hazard ratios (HRs) with 95% confidence intervals (CIs) were reported.

Air pollution variables (NO_2_, PM_10_, PM_2.5_) were modeled as continuous variables using clinically interpretable scaling (per 10 µg/m^3^ for NO_2_ and PM_10_, per 5 µg/m^3^ for PM_2.5_). Both single-pollutant and multi-pollutant models were constructed to account for potential collinearity. Scaling (per 10 µg/m^3^ for NO_2_/PM_10_ and 5 µg/m^3^ for PM_2.5_) was chosen to reflect distribution ranges and ensure clinically interpretable hazard ratios.

In multivariable analysis, the proportional hazards assumption was verified using Schoenfeld residuals; no significant violations were observed.

Non-linearity was explored using restricted cubic splines; no meaningful deviations from linearity were observed, supporting the use of linear terms. Non-linearity was explored using restricted cubic splines (three knots); visual inspection and model fit comparisons did not indicate meaningful deviation from linearity, supporting the use of linear terms.

### 2.6. Model Construction and Variable Selection

Variables included in the multivariable model were selected a priori based on clinical relevance and prior literature, rather than automated stepwise procedures. The final multivariable Cox model included 11 predictors for 220 observed deaths, yielding approximately 20 events per variable, within conventionally accepted thresholds for survival modeling.

All candidate variables were first evaluated in univariable analysis. Variables demonstrating clinical relevance or statistical significance (*p* < 0.10) were considered for inclusion.

To avoid multicollinearity, variance inflation factors (VIF) were calculated. Variables demonstrating collinearity (VIF > 3) were excluded from the final model. Specifically, renal impairment was excluded from the final multivariable Cox model due to collinearity with other covariates, despite being significant in univariable analysis, but still analyzed predictively. Calibration of the final model was assessed using the bootstrap-corrected calibration slope and Brier score.

Model discrimination was assessed using time-dependent receiver operating characteristics (ROC) to account for censoring, with area under the curve (AUC) reported along with 95% confidence intervals. Time-dependent ROC curves were computed using inverse probability-of-censoring weighting. Differences in AUC between models were evaluated using bootstrap resampling (1000 iterations) with percentile-based confidence intervals. Moreover, time-dependent ROC methodology was applied to account for censored survival data. Predicted risks were derived from Cox model-based survival probabilities, and inverse probability-of-censoring weighting was used for discrimination analyses.

To evaluate incremental prognostic value, net reclassification improvement (NRI) and integrated discrimination improvement (IDI) were calculated. Net reclassification improvement (NRI) was calculated using the continuous (category-free) approach, as predefined clinically meaningful risk categories for long-term mortality in this population are not established. Continuous NRI quantifies correct directional changes in predicted risk; however, it may overestimate improvement and should be interpreted cautiously. NRI quantified the proportion of individuals correctly reclassified into higher or lower risk categories, while IDI assessed improvement in separation between predicted risks for events and non-events.

The events-per-variable ratio in the primary multivariable model remained within conventionally accepted ranges for Cox regression modeling, reducing but not eliminating the possibility of overfitting.

### 2.7. Methodology of Decision Curve Analysis

To evaluate the clinical utility of the prediction models, decision curve analysis (DCA) was performed by quantifying the net benefit across a range of clinically relevant threshold probabilities (0.01–0.50). Net benefit was calculated as the difference between the proportion of true positives and the proportion of false positives weighted by the relative harm of false-positive classifications, according to the standard formulation: net benefit = (true positives/N) − (false positives/N) × (threshold probability/(1 − threshold probability)). The clinical model and the combined clinical-plus-pollution model were compared against default strategies (treat-all and treat-none). This approach allows assessment of whether the inclusion of environmental exposure variables provides meaningful improvement in clinical decision-making across varying risk thresholds.

Optimal thresholds for NO_2_ and PM_10_ were determined using the Youden index, and these thresholds were used for Kaplan–Meier survival analyses. Survival curves were compared using the log-rank test, and hazard ratios were estimated using Cox regression.

To stratify patients by multivariable risk, predicted probabilities from the clinical and clinical-plus-pollution models were dichotomized at the median, and Kaplan–Meier curves were constructed accordingly. In detail, two approaches to risk stratification were used: (1) pollutant-specific thresholds derived from the Youden index and (2) model-based stratification using median predicted risk.

Given the number of covariates relative to the number of events, potential overfitting and multicollinearity were considered. Model stability was assessed by the consistency of effect estimates across different model specifications.

All statistical analyses were performed using Python (version 3.11) with the following libraries: pandas for data handling, NumPy for numerical computations, scikit-learn for predictive modeling and receiver operating characteristic (ROC) analysis, statsmodels for survival analyses, including Cox proportional hazards modeling, and Matplotlib version 3.7.5 for graphical visualization.

Reclassification metrics, including net reclassification improvement (NRI) and integrated discrimination improvement (IDI), were calculated using custom Python implementations based on established methodological definitions.

All tests were two-sided, and *p*-values < 0.05 were considered statistically significant.

## 3. Results

The multicenter retrospective analysis included 1033 patients who underwent surgery at two cardiac surgery centers between 2006 and 2025 and were followed up for 8.1 (3.4–11.1) years. There were 813 patients (survival group) and 220 patients (deceased group). The median age was 58 (52–64) years, and off-pump surgery was performed in 47.6% (492) patients. All analyzed patients underwent an operation with two internal mammary artery grafts. The detailed information, including demographic and clinical characteristics, followed by perioperative data, was presented in Table 1.

### 3.1. Univariate and Multivariable Analysis for Mortality Risk Prediction

In the performed models, among clinical covariates, older age (HR 1.08 per year; *p* < 0.001), lower left ventricular ejection fraction (HR 0.98 per 1% increase; *p* < 0.001), diabetes mellitus (HR 1.65; *p* = 0.015), peripheral vascular disease (HR 1.78; *p* = 0.003), prior cerebrovascular events (HR 4.24; *p* < 0.001), and severe renal impairment (HR 2.11; *p* = 0.006) were associated with increased mortality. Conversely, a higher number of grafts (HR 0.74; *p* = 0.018) and the off-pump CABG (OPCAB) technique (HR 0.67; *p* = 0.028) were associated with reduced mortality risk. The significant impact of exposure to air pollutants (NO_2_ and PM_10_) on long-term mortality was presented (Table 2).

Internal validation using bootstrap resampling demonstrated stable discrimination estimates, with bootstrap internal validation using 1000 resamples demonstrating limited optimism, with optimism-corrected AUC decreasing from 0.84 to 0.82, supporting the model’s overall robustness despite the observational design and the moderate event count. The bootstrap-corrected calibration slope was 0.94, and the Brier score was 0.16, suggesting acceptable calibration.

### 3.2. Multipollutant Models

To assess the effects of individual pollutants while accounting for co-exposure, multipollutant Cox proportional hazards models were constructed simultaneously for NO_2_, PM_10_, and PM_2.5_.

Pearson correlation analysis demonstrated moderate correlations among pollutants, particularly between NO_2_ and PM_10_ (r = 0.58–0.65, *p* < 0.001), supporting partial shared exposure patterns without evidence of prohibitive collinearity.

In these models, both NO_2_ and PM_10_ remained significantly associated with increased mortality, although effect sizes were modestly attenuated compared to single-pollutant models, suggesting partial collinearity between pollutants.

Specifically:

- NO_2_ remained associated with mortality (HR 2.21, 95% CI 1.60–3.05; *p* < 0.001).
- PM_10_ remained significant (HR 2.09, 95% CI 1.42–3.08; *p* < 0.001).
- PM_2.5_ remained non-significant (HR 1.08, 95% CI 0.85–1.39; *p* = 0.52).

Variance inflation factor (VIF) analysis indicated moderate collinearity (VIF range: 2.1–3.4), but below the thresholds for model instability, suggesting model stability as presented in Table 3.

### 3.3. Air-Pollution Results

In multivariable Cox proportional hazards analysis, adjusting for demographic, clinical, and procedural characteristics, both nitrogen dioxide (NO_2_) and particulate matter ≤ 10 µm (PM_10_) were associated with increased long-term mortality. Specifically, each 10 µg/m^3^ increase in NO_2_ was associated with a significantly higher risk of mortality (hazard ratio [HR] 2.70, 95% confidence interval [CI] 2.03–3.59; *p* < 0.001). Similarly, each 10 µg/m^3^ increase in PM_10_ was associated with increased mortality risk (HR 2.73, 95% CI 1.94–3.83; *p* < 0.001). In contrast, particulate matter ≤ 2.5 µm (PM_2.5_) was not significantly associated with long-term mortality in the adjusted model (per 5 µg/m^3^: HR 1.20, 95% CI 0.96–1.50; *p* = 0.102).

### 3.4. ROC Discrimination Analysis

Receiver operating characteristic analysis was performed to assess discrimination for long-term mortality. The baseline clinical model, including age, sex, BMI, hypertension, diabetes mellitus, hyperlipidemia, renal impairment, peripheral vascular disease, prior cerebrovascular events, urgency status, OPCAB, number of grafts, and left ventricular ejection fraction, demonstrated moderate discrimination for the combined clinical model (AUC 0.73, 95% CI 0.69–0.77).

The addition of NO_2_ and PM_10_ substantially improved discrimination, with the clinical-plus-pollution model reaching an AUC of 0.84 (95% CI 0.81–0.87). When considered separately, the strongest single-variable discrimination was observed for NO_2_ (AUC 0.75, 95% CI 0.71–0.78) and PM_10_ (AUC 0.70, 95% CI 0.66–0.73), whereas individual clinical variables showed only modest discrimination, with AUCs ranging from 0.51 to 0.61.

The predicted ROC analyses for clinical and environmental long-term mortality risk factors were presented in Table 4.

**Figure 2:**
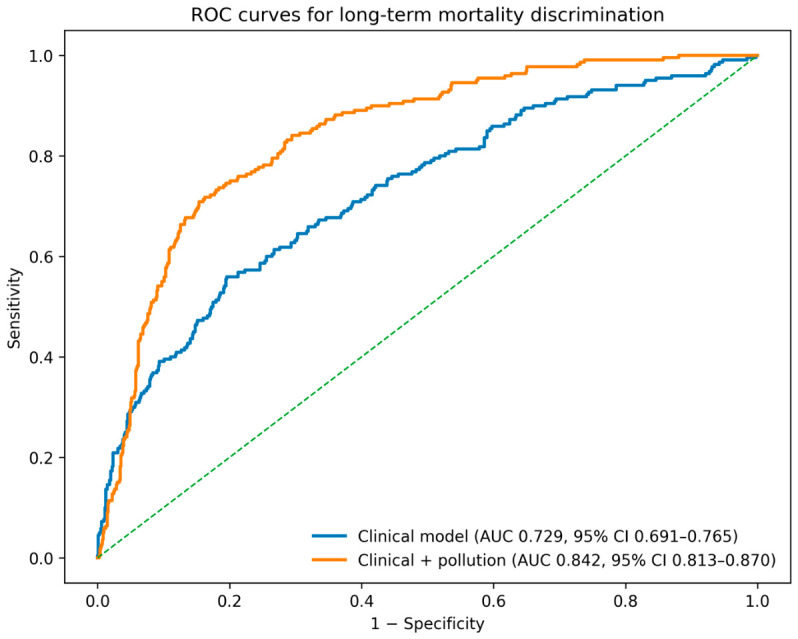
Receiver operating characteristic (ROC), including area under the curve (AUC) and 95% confidence intervals (95% CI), analysis for clinical and clinical + pollution factors in long-term mortality prediction. The green line means: pollution factors in long-term mortality prediction.

Receiver operating characteristic (ROC) curves for the clinical factors (age, LVEF, diabetes, PVD, prior CVAEs, and renal impairment) were compared with ROC curves for clinical factors and pollution compounds (PM_10_ + NO_2_), as shown in Figure 2.

### 3.5. Youden Index Thresholds

**Figure 3:**
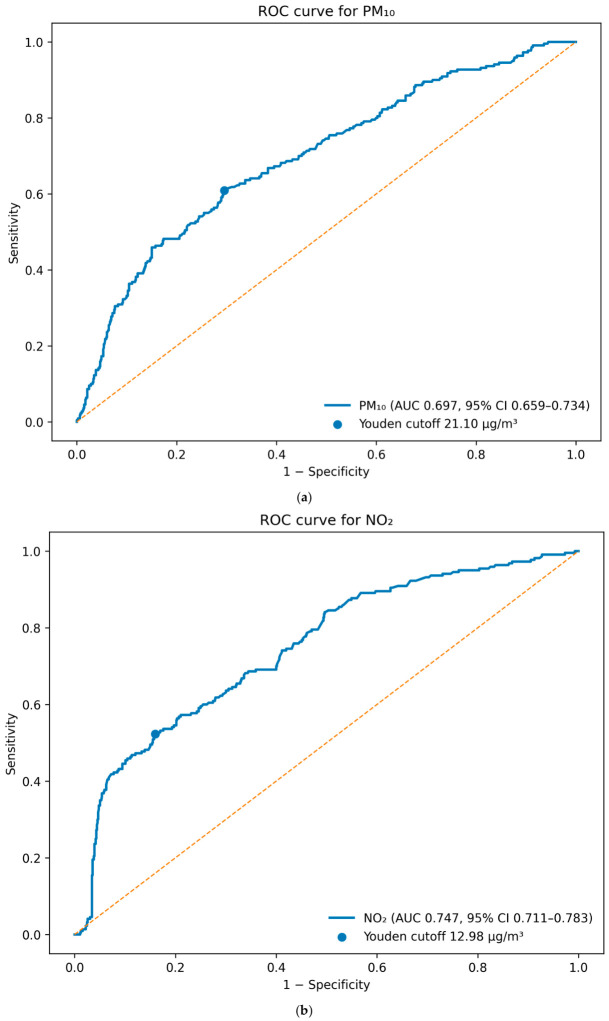
(**a**) Receiver operating characteristic (ROC) analysis (including area under the curve value (AUC) and 95 % confidence intervals (95% CI)) of PM_10_’s impact on long-term mortality prediction, with a calculated Youden cut-off value. (**b**) Receiver operating characteristic (ROC) analysis (including area under the curve value (AUC) and 95 % confidence intervals (95% CI)) of NO_2_ impact on long-term mortality prediction, with a calculated Youden cut-off value.

The optimal NO_2_ threshold identified by the Youden index was 12.98 µg/m^3^, corresponding to a sensitivity of 52.3% and a specificity of 84.0%. The optimal PM_10_ threshold was 21.10 µg/m^3^, corresponding to a sensitivity of 60.9% and a specificity of 70.4%, as shown in Figure 3a,b. These thresholds were used for subsequent Kaplan–Meier stratification.

Schoenfeld residual testing showed no significant violations of the proportional hazards assumption (global test *p* > 0.10).

### 3.6. Kaplan–Meier Analysis

**Figure 4:**
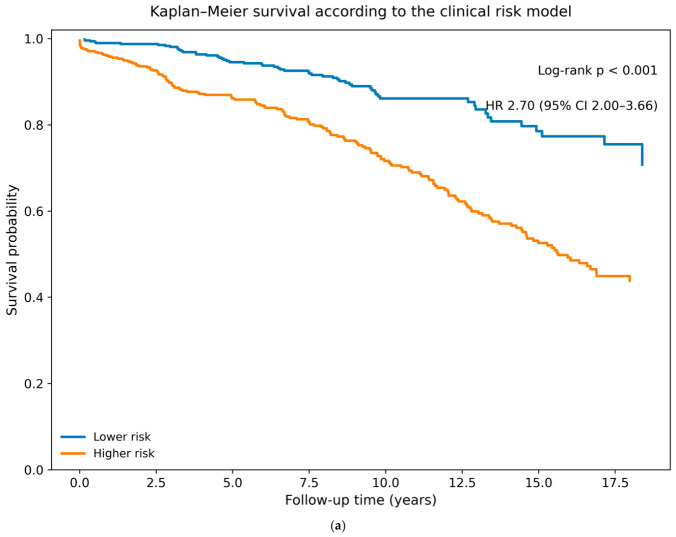

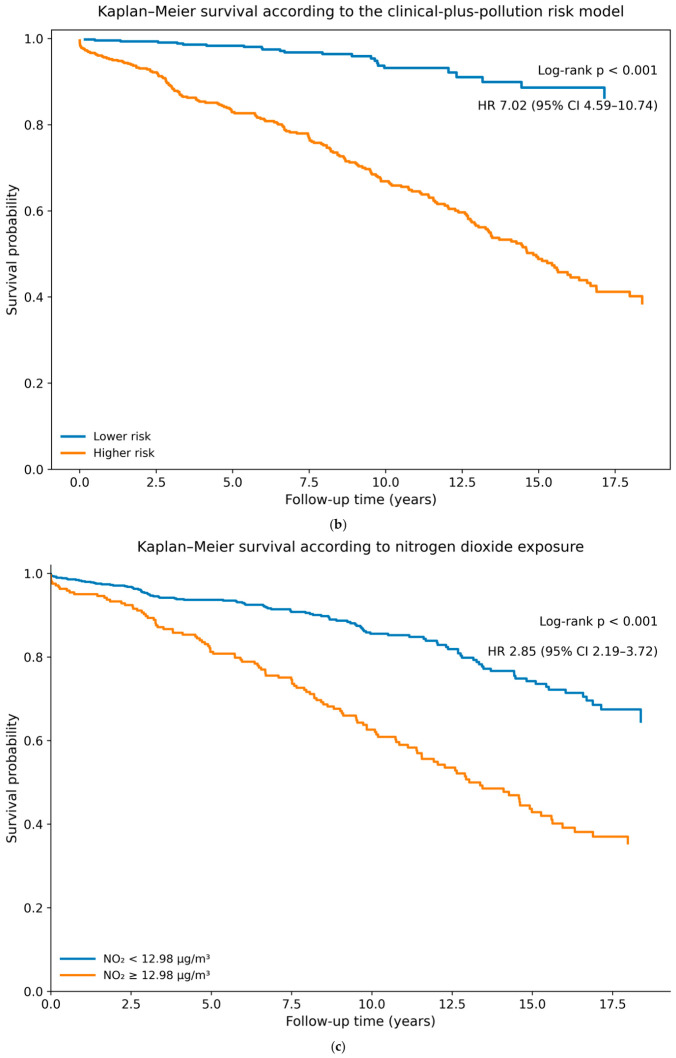

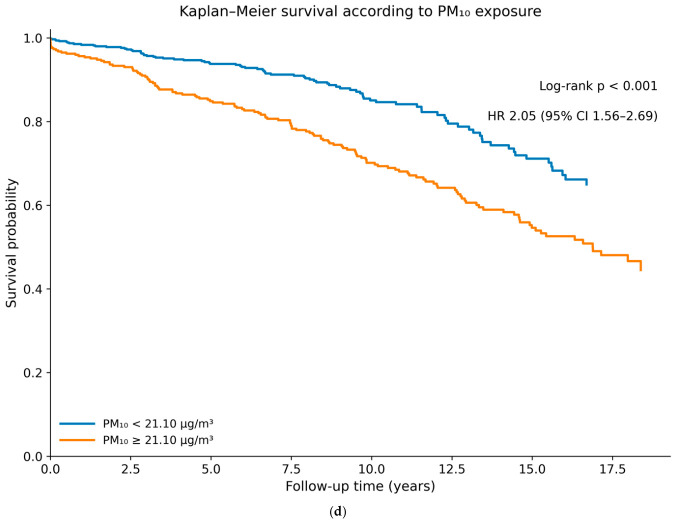
(**a**) Kaplan–Meier survival curves by clinical factors. (**b**) Kaplan–Meier survival curves by clinical and air pollution factors. (**c**) Kaplan–Meier survival curves by nitrogen dioxide exposure with -a presented a cut-off value of 12.98 µg/m^3^. (**d**) Kaplan–Meier survival curves by PM_10_ exposure, with a presented cut-off value of 21.10 µg/m^3^.

The Kaplan–Meier curves for long-term mortality prediction, stratified by the analyzed clinical and environmental factors, were constructed. The clinical model alone stratified patients into significantly different risk groups (HR 2.70, 95% CI 2.00–3.66; log-rank *p* < 0.001), with further improvement observed after inclusion of environmental exposure variables (Figure 4a,b).

The Kaplan–Meier analysis showed clear separation of the long-term survival curves above and below the Youden-derived pollutant thresholds. For NO_2_, exposure at or above 12.98 µg/m^3^ was associated with significantly worse survival than lower exposure, with a log-rank *p*-value of <0.001 and an unadjusted hazard ratio of 2.85 (95% CI 2.19–3.72) (Figure 4c).

For PM_10_, exposure at or above 21.10 µg/m^3^ was likewise associated with significantly reduced long-term survival, with a log-rank *p*-value of <0.001 and an unadjusted hazard ratio of 2.05 (95% CI 1.56–2.69), as presented in Figure 4d.

The comparison of receiver operating characteristic (ROC) and Kaplan–Meier (KM) curves for clinical factors, combined clinical and pollution factors, and lone PM_10_ and NO_2_ exposure is presented in Table 5.

### 3.7. Incremental Prognostic Value

Adding environmental exposure variables to the clinical model significantly improved risk classification. Net reclassification improvement (NRI) was 0.42 (*p* < 0.001), indicating that 42% of patients were more accurately reclassified with respect to mortality risk.

Similarly, integrated discrimination improvement (IDI) was 0.11 (*p* < 0.001), demonstrating a substantial increase in the separation of predicted risk between patients who died and those who survived.

These findings confirm that air pollution exposure provides meaningful incremental prognostic information beyond traditional clinical risk factors.

### 3.8. Decision Curve Analysis

Decision curve analysis demonstrated that the clinical-plus-pollution model provided consistently greater net benefit than the clinical model alone, as well as the default treat-all and treat-none strategies, across the full range of examined threshold probabilities (0.01–0.50).

**Figure 5:**
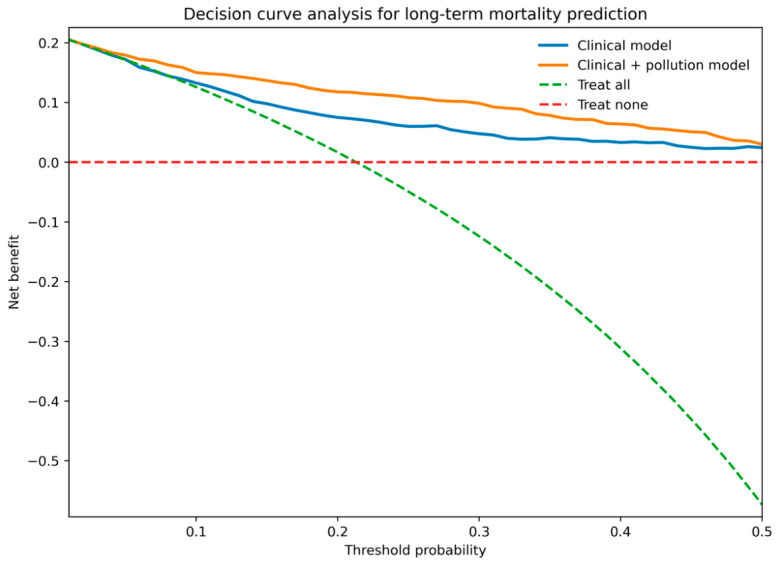
Decision curve analysis for long-term mortality prediction.

At selected threshold probabilities, the incremental net benefit of the clinical-plus-pollution model over the clinical model was: 0.05: +0.0071, 0.10: +0.0175, 0.15: +0.0385, 0.20: +0.0429, 0.25: +0.0481, 0.30: +0.0509. The advantage becomes more pronounced as the threshold probability increases; the maximum incremental net benefit was observed at thresholds between 0.20 and 0.30, as presented in Figure 5.

The incremental net benefit associated with including environmental exposure increased progressively at higher threshold probabilities, indicating improved clinical utility of the combined model in identifying patients at elevated long-term risk.

These findings support the relevance of incorporating air pollution exposure into prognostic assessment beyond conventional clinical risk factors.

### 3.9. Sensitivity Analyses

Several sensitivity analyses were conducted to assess the robustness of the findings (Table 6).

1. Alternative Model Specifications

Results remained consistent after excluding variables with borderline significance and including all clinically relevant covariates (full model).

2. Non-linear Effects

Restricted cubic spline analysis did not demonstrate substantial non-linearity for NO_2_ or PM_10_, supporting the use of linear modeling assumptions.

3. Proportional Hazards Assumption

**Figure 6:**
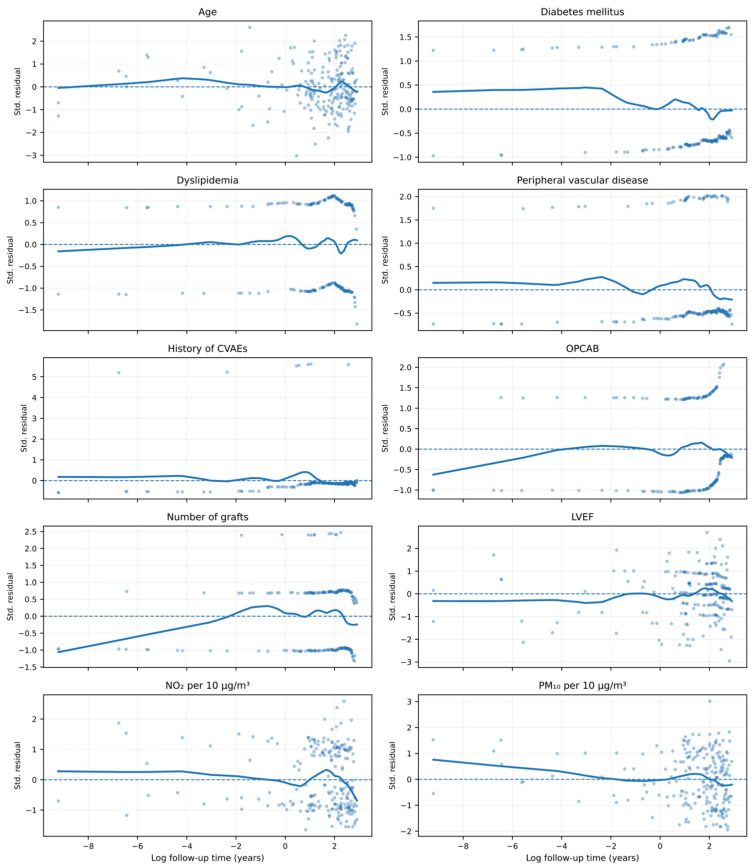
Schoenfeld residual assessment of the proportional hazards assumption. Standardized Schoenfeld residuals are plotted against log follow-up time for covariates included in the multivariable Cox model. The global test showed no significant violation of the proportional hazards assumption (*p* = 0.155). Abbreviations: LVEF—left ventricular ejection fraction, m^3^—cubic meter, NO_2_—nitrogen dioxide, OPCAB—off-pump coronary artery bypass grafting, PM_10_—with an aerodynamic diameter ≤ 10 μm, μg—microgram.

Schoenfeld residual testing confirmed no significant violations of the proportional hazards assumption (global test *p* > 0.10) as presented in Figure 6.

4. Exclusion of Early Deaths

After excluding deaths within the first 12 months (n = 26), results remained materially unchanged.

5. Alternative Exposure Window

Analyses using baseline exposure (first-year exposure only) yielded consistent directions and magnitudes of association.

6. Model Stability

Bootstrap resampling (1000 iterations) confirmed the stability of hazard ratio estimates and model discrimination metrics.

Collectively, these analyses support the robustness of the association between long-term exposure to NO_2_ and PM_10_ and mortality.”

## 4. Discussion

The present study demonstrates that long-term exposure to NO_2_ and PM_10_ is associated with increased mortality following CABG, even after adjustment for an extensive set of clinical and procedural variables [28]. Importantly, including environmental exposure in a comprehensive clinical model led to consistent improvements across discrimination, reclassification, and decision-analytic metrics, indicating that these associations translate into meaningful differences in risk stratification.

The convergence of multiple analytical approaches strengthens the robustness of the findings. Environmental exposure remained significant in multivariable Cox regression, improved model discrimination (ΔAUC 0.11), enhanced patient reclassification (NRI 0.42; IDI 0.11), and demonstrated superior net clinical benefit in decision curve analysis. Together, these results suggest that air pollution provides incremental prognostic information beyond traditional risk factors rather than simply acting as a correlate of baseline risk. Although NRI suggested substantial improvement in risk classification, this metric should be interpreted in the context of its known methodological limitations, particularly the tendency of continuous NRI to overestimate incremental value. For this reason, NRI results were interpreted as supportive rather than definitive and were considered alongside discrimination and decision-analytic measures. The Youden-derived thresholds should be interpreted primarily as exploratory tools for visualization and risk stratification rather than clinically actionable cut-points. Dichotomization of continuous exposure variables may reduce statistical efficiency and introduce instability in threshold estimation, particularly in the absence of external validation.

These findings extend existing literature by focusing on a surgically revascularized population. While CABG addresses epicardial coronary obstruction, it does not modify systemic processes such as inflammation, oxidative stress, or endothelial dysfunction. Persistent environmental exposure may therefore continue to influence vascular biology and long-term outcomes despite successful revascularization. Our results challenge the conventional paradigm in which long-term outcomes after CABG are predominantly explained by patient-level characteristics [29,30,31,32]. While revascularization addresses epicardial coronary obstruction, it does not eliminate the systemic processes that drive cardiovascular progression. In this context, ambient air pollution may act as a chronic external stimulus that sustains vascular injury and promotes adverse remodeling, thereby influencing prognosis independently of surgical success [33]. The magnitude of the observed hazard ratios substantially exceeds that reported in most population-based environmental epidemiology studies and therefore should be interpreted cautiously. It is plausible that the estimated effect sizes partially reflect residual confounding, contextual vulnerability, exposure misclassification, or clustering of higher-risk patients within more polluted environments rather than a purely direct biological effect of pollution exposure.

Our analysis was focused on a unique subgroup of CAD patients, those who were characterized by multivessel disease and referred for surgical revascularization. The potential impact of air pollution was higher than in previous reports [34], indicating a greater environmental burden in the surgical cohort, which can be explained by less advanced coronary atherosclerosis. In our analyses [22], premature coronary disease progression, as indicated by a calculated HR of 2.0, was associated with nitrogen dioxide exposure. In the Lui et al. [35] analysis, a 1.69-fold increase in coronary disease progression was associated with a 10 µg/m^3^ increase in PM_10_ exposure. Several factors may contribute to this discrepancy, including residual confounding, exposure misclassification, clustering of higher-risk individuals in more polluted regions, model instability, and the potentially greater vulnerability of surgically treated patients with advanced coronary disease. Therefore, the absolute magnitude of the hazard ratios should be interpreted cautiously. The principal strength of the findings lies in the consistency and direction of the observed associations across multiple analytical approaches rather than the exact numerical size of the estimated effects.

Multipollutant models confirmed that NO_2_ and PM_10_ exert effects, supporting the robustness of the findings despite moderate collinearity between exposure variables.

Decision curve analysis demonstrated that incorporating environmental exposure provides incremental clinical utility across a range of threshold probabilities, particularly between 0.20 and 0.30, where the net benefit gain was most pronounced. This suggests that including air pollution metrics may improve the identification of patients at intermediate-to-high risk, where clinical decision-making is most uncertain.

Nitrogen dioxide and coarse particulate matter are known to induce oxidative stress, endothelial dysfunction, and activation of inflammatory signaling pathways [36,37,38]. These processes contribute to impaired vascular repair, increased arterial stiffness, and a prothrombotic milieu. In patients following CABG, such mechanisms may accelerate graft failure, promote microvascular dysfunction, or exacerbate non-coronary cardiovascular disease, ultimately translating into increased mortality risk. Notably, the absence of a statistically robust association for PM_2.5_ in the fully adjusted model suggests that not all pollutant fractions exert equivalent effects in this clinical context [39,40]. This may reflect differences in particle composition, exposure patterns, or collinearity between pollutants, rather than a true absence of biological relevance. The consistency of the direction of effect across models, however, supports a broader signal linking air pollution to adverse outcomes.

The magnitude of the observed associations is notably higher than that reported in most population-based studies, where effect sizes for similar pollutant increments are typically modest. This discrepancy may reflect differences in study design, including the use of residential-level exposure estimates, which may introduce exposure misclassification, and the potential clustering of high-risk patients in more polluted regions. Additionally, residual confounding and collinearity between pollutants may have contributed to the inflation of effect estimates. Therefore, the absolute magnitude of the hazard ratios should be interpreted cautiously, and emphasis should be placed on the direction and consistency of the associations rather than their exact size.

Importantly, the magnitude of the observed hazard ratios should not be interpreted as evidence of a direct causal effect. Given the observational design and the potential for residual and unmeasured confounding, these estimates are more appropriately viewed as reflecting the combined influence of environmental exposure and correlated contextual factors. Therefore, the strength of the present findings lies primarily in the consistency and direction of associations across multiple analytical approaches rather than the absolute size of the effect estimates.

In contrast to NO_2_ and PM_10_, PM_2.5_ was not associated with mortality in adjusted analyses. This finding differs from several prior population-based studies and may reflect collinearity between pollutants, regional differences in pollutant composition, exposure misclassification, or limited statistical power to detect smaller independent effects. Accordingly, the present findings should not be interpreted as evidence that PM_2.5_ lacks biological relevance, but rather that an association could not be demonstrated within the current analytical framework. The absence of an association between PM_2.5_ and cardiovascular mortality contrasts with several population-based studies that have consistently linked particulate matter to cardiovascular mortality [41,42,43].

From a clinical perspective, these findings do not imply immediate changes in patient management but suggest that environmental exposure may represent an additional dimension of risk stratification. Incorporating such variables into predictive models may improve the identification of higher-risk patients; however, these observations require external validation and prospective investigation before clinical implementation can be considered.

Traditionally, long-term prognosis after CABG has been conceptualized as primarily determined by intrinsic patient factors and procedural success. However, our findings suggest that this paradigm is incomplete. Even after surgical correction of coronary anatomy, patients remain exposed to persistent systemic stimuli that may influence vascular biology and long-term outcomes. The improvement in discrimination (ΔAUC 0.11), combined with strong NRI and IDI values, suggests that environmental exposure is not only statistically significant but also clinically actionable in risk stratification. These findings support further investigation into whether environmental exposure metrics may improve future prognostic models.

In this context, air pollution may act as a persistent environmental exposure associated with adverse cardiovascular risk profiles. The magnitude of its impact, as demonstrated by both hazard ratios and discrimination metrics, suggests that environmental exposure may represent an additional dimension of long-term cardiovascular risk that warrants consideration alongside traditional clinical factors. These findings support a broader conceptual shift in cardiovascular medicine. Rather than viewing risk as confined to patient-specific characteristics, it may be more appropriate to consider it as a dynamic interaction between the individual and their environment. In this framework, CABG represents a modification of anatomical disease, but not of the external factors that continue to influence vascular health. The present findings suggest that environmental exposure may contribute to long-term cardiovascular risk even after anatomically successful revascularization.

Given the study’s observational, retrospective design, causal inference cannot be established. The observed associations should be interpreted as hypothesis-generating and reflective of potential relationships rather than definitive evidence of causality.

### Limitations

This study has several limitations. First, its observational design precludes causal inference, and residual confounding cannot be excluded. Second, environmental exposure was estimated at the regional level and may not fully reflect individual-level exposure variability. Third, the number of covariates relative to the number of events raises the possibility of model overfitting, although consistency across multiple analytical approaches supports the robustness of the findings. Residual confounding represents the most important limitation of the present analysis. Several variables strongly associated with both residential pollution exposure and cardiovascular mortality—including smoking history, socioeconomic deprivation, educational status, occupational exposure, medication adherence, and lifestyle-related behaviors—were unavailable and therefore could not be incorporated into the multivariable models. In environmental epidemiology, residential exposure may partly function as a surrogate marker of broader contextual vulnerability. Consequently, a portion of the observed association may reflect correlated social and behavioral determinants rather than a direct biological effect of pollution exposure. Finally, all analyses were internally derived and require external validation to confirm generalizability.

The authors acknowledge that residual confounding by smoking and other unmeasured lifestyle or socioeconomic factors may partly explain the observed associations and the magnitude of the effect estimates.

Exposure estimates were based on residential-level data and may not fully capture individual variability, including occupational exposure, indoor air quality, or temporal fluctuations.

The use of data-derived thresholds (Youden index) without external validation may introduce optimism bias due to overfitting, and results from Kaplan–Meier stratification should therefore be interpreted cautiously. Although multiple sensitivity analyses confirmed robustness, residual confounding cannot be excluded.

Socioeconomic status, meteorological factors (e.g., temperature, humidity), and occupational exposures were unavailable and may act as important confounders, as they are associated with both residential pollution exposure and cardiovascular outcomes. Their omission may have led to residual confounding and potential overestimation of observed associations. These factors may partially explain the observed associations and should be addressed in future studies.

We acknowledge that residual confounding cannot be excluded and may be substantial (socioeconomic status, smoking history, occupational exposures, and urban–rural differences were not available and could not be incorporated into the models). These factors are known to be associated both with environmental pollution exposure and cardiovascular outcomes, and their omission may have influenced the observed associations. As a result, the findings should be interpreted as associative rather than causal, and the possibility that part of the observed effect reflects unmeasured confounding must be acknowledged.

Cause-specific mortality data were not consistently available across participating centers, and therefore, cardiovascular mortality analyses could not be performed.

In addition, all model development, threshold derivation, and performance assessment were conducted within the same dataset. Although internal validation using bootstrap resampling demonstrated stability of the estimates, the absence of external validation limits the generalizability of the findings and introduces the possibility of optimistic bias. Future studies should confirm these results in large-volume cohorts.

## 5. Conclusions

In this multicenter retrospective cohort of patients undergoing CABG with BIMA grafting, higher long-term residential exposure to NO_2_ and PM_10_ was associated with increased all-cause mortality after adjustment for measured clinical and procedural factors. Environmental exposure variables improved statistical risk discrimination and reclassification; however, the observational design and potential for substantial residual confounding preclude causal inference. These findings support further investigation of environmental exposure as a possible prognostic marker in surgically treated coronary artery disease, pending external validation and more comprehensive adjustment for contextual and behavioral confounders.

## Figures and Tables

**Table 1:** Patients characteristics.

Variables	Analyzed Sample n = 1033	Survival Group (1) n = 813	Deceased Group (2) n = 220	*p*-Value 1 vs. 2
Demographical				
Age (years) (median (Q1–Q3))	58 (52–64)	58 (52–63)	61 (55–68)	<0.001
2. Sex (female) (n (%))	148 (14.3)	114 (14.0)	34 (15.5)	0.591
3. BMI (kg/m^2^) (median (Q1–Q3))	27.8 (25.6–30.5)	27.9 (25.7–30.6)	27.5 (24.9–30.0)	0.125
Co-morbidities				
Arterial hypertension (n (%))	920 (89.1)	724 (89.1)	196 (89.1)	0.987
2. Diabetes mellitus (n (%))	251 (24.3)	182 (22.4)	69 (31.4)	0.006
3. Dyslipidemia (n (%))	500 (48.4)	385 (47.4)	115 (52.3)	0.196
4. Kidney impairment * (n (%))	522 (50.5)	378 (46.5)	144 (65.5)	<0.001
5. PVD (n (%))	123 (11.9)	77 (9.5)	46 (20.9)	<0.001
6. History of CVAEs (n (%))	21 (2.0)	14 (2.0)	7 (3.0)	0.174
Surgical characteristics				
Urgent/emergency (n (%))	498 (48.2)	396 (48.7)	102 (46.4)	0.537
2. OPCAB (n (%))	492 (47.6)	419 (51.5)	73 (33.2)	<0.001
3. Number of grafts (mean (SD))	2.6 (0.6)	2.7 (0.6)	2.6 (0.6)	0.350
Echocardiography:				
LVEF (%) (median (Q1–Q3))	55 (49–60)	55 (50–60)	50 (45–60)	<0.001

Abbreviations: BMI—body mass index, CVAE—cardiovascular adverse events, OPCAB—off-pump coronary artery bypass grafting, LVEF—left ventricular ejection fraction, n—number, PVD—peripheral artery disease, SD—standard deviation, Q—quartile. * defined as GFR below 60 mL/min/1.73 m^2^.

**Table 2:** Univariate and multivariable models for long-term mortality prediction.

Variable	Univariable			Multivariable		
	HR	HR (95% CI)	*p*-Value	HR	HR (95% CI)	*p*-Value
Age (per year)	1.08	1.07–1.10	<0.001	1.07	1.05–1.09	<0.001
Female sex	1.21	0.84–1.75	0.298	--	--	--
BMI (per kg/m^2^)	0.99	0.95–1.03	0.581	--	--	--
Hypertension	1.35	0.88–2.07	0.164	--	--	--
Diabetes mellitus	2.08	1.56–2.77	<0.001	1.87	1.37–2.54	<0.001
Dyslipidemia	1.27	0.97–1.66	0.078	1.35	1.02–1.78	0.034
Renal impairment	1.53	1.16–2.02	0.003	--	--	--
Peripheral vascular disease	2.51	1.81–3.47	<0.001	1.84	1.29–2.61	<0.001
History of CVAEs	2.65	1.25–5.63	0.011	2.91	1.33–6.40	0.008
Urgent/emergent surgery	1.25	0.95–1.63	0.105	--	--	--
OPCAB	0.94	0.70–1.26	0.675	0.71	0.52–0.99	0.042
Number of grafts	0.99	0.79–1.23	0.895	0.75	0.58–0.95	0.020
LVEF (per 1%)	0.96	0.95–0.98	<0.001	0.97	0.96–0.98	<0.001
NO_2_ (per 10 µg/m^3^)	2.22	1.77–2.79	<0.001	2.70	2.03 to 3.59	<0.001
PM_10_ (per 10 µg/m^3^)	2.27	1.71–3.02	<0.001	2.73	1.94 to 3.83	<0.001

Abbreviations: BMI, body mass index; CI, confidence interval; CVAE, cerebrovascular adverse event; HR, hazard ratio; LVEF, left ventricular ejection fraction; NO_2_, nitrogen dioxide; OPCAB, off-pump coronary artery bypass; PM_10_, particulate matter ≤ 10 μm.

**Table 3:** Multipollutant models account for collinearity among exposure variables.

Variable	HR	95% CI	*p*-Value
NO_2_ (per 10 µg/m^3^)	2.21	1.60–3.05	<0.001
PM_10_ (per 10 µg/m^3^)	2.09	1.42–3.08	<0.001
PM_2.5_ (per 5 µg/m^3^)	1.08	0.85–1.39	0.52

Abbreviations: CI—confidence interval, HR—hazard ratio, m^3^—cubic meter, NO_2_—nitrogen dioxide, PM_2.5_—particulate matter with an aerodynamic diameter ≤ 2.5 μm, PM_10_—with an aerodynamic diameter ≤ 10 μm, μg—microgram.

**Table 4:** Receiver operating characteristic curves for clinical and environmental factors.

Variable		Cut-Off Value	AUC	95% CI	Sensitivity	Specificity
Combined clinical factors		---	0.73	0.69–0.77		
Clinical factors analyzed separately	Age	68 years	0.72	0.69–0.75	0.26	0.89
	LVEF	56%	0.70	0.67–0.73	0.06	0.96
	Diabetes	1 (yes)	0.60	0.57–0.63	0.31	0.78
	PVD	1 (yes)	0.62	0.59–0.65	0.21	0.91
	Prior CVAEs	1 (yes)	0.58	0.55–0.61	0.03	0.98
	Renal impairment	1 (yes)	0.61	0.58–0.64	0.65	0.54
Combined air pollution factors		---	0.79	0.75–0.82		
Air pollution factors analyzed separately	NO_2_	12.98 µg/m^3^	0.75	0.71–0.78	0.52	0.84
	PM_10_	21.10 µg/m^3^	0.70	0.66–0.73	0.61	0.70

Abbreviations: CVAE—cardiovascular adverse events, LVEF—left ventricular ejection fraction, NO_2_—nitrogen dioxide, PM—particulate matter, PVD—peripheral artery disease.

**Table 5:** ROC and Kaplan–Meier characteristics for long-term survival after CABG procedures.

Model	AUC	95% CI	Youden	Sensitivity	Specificity	KM Log	KM HR	KM 95% CI
Clinical	0.73	0.70–0.77	-	-	-	<0.001	2.7	2.00–3.66
Clinical + pollution	0.84	0.81–0.87	-	-	-	<0.001	7.02	4.56–10.74
NO_2_	0.75	0.71–0.78	12.98	0.52	0.84	<0.001	2.85	2.19–3.72
PM_10_	0.70	0.66–0.73	21.1	0.61	0.70	<0.001	2.05	1.56–2.68

Abbreviations: AUC—area under the curve, CI—confidence interval, HR—hazard ratio, KM—Kaplan–Meier, NO_2_—nitrogen dioxide, PM_10_—particulate matter up to 10 microns.

**Table 6:** Sensitivity results.

Analysis	NO_2_ HR	PM_10_ HR	Interpretation
Base model	2.70	2.73	Main result
Excluding early deaths	2.52	2.61	Stable
Full covariate model	2.64	2.68	Consistent
Baseline exposure only	2.40	2.55	Direction preserved

Abbreviations: HR—hazard ratio, NO_2_—nitrogen dioxide, PM_10_—with an aerodynamic diameter ≤ 10 μm.

## Data Availability

Data supporting the reported results are available upon reasonable request to the corresponding authors. The restriction is related to privacy or ethical restrictions.
